# Real-world treatment patterns in patients with nontuberculous mycobacterial lung disease in the Netherlands based on medication dispensing data

**DOI:** 10.1186/s12890-023-02460-1

**Published:** 2023-06-20

**Authors:** W Hoefsloot, E Dacheva, R van der Laan, M Krol, J van Ingen, M Obradovic, Ximeng Liu

**Affiliations:** 1 Radboudumc Center for Infectious Diseases, Department of Pulmonary Diseases, Radboud University Medical Center, Nijmegen, Netherlands; 2 IQVIA, Amsterdam, Netherlands; 3 Insmed Netherlands B.V, Utrecht, Netherlands; 4 Radboudumc Center for Infectious Diseases, Department of Medical Microbiology, Radboud University Medical Center, Nijmegen, Netherlands; 5 Insmed Germany GmbH, Frankfurt am Main, Germany

**Keywords:** Nontuberculous mycobacterial lung disease, Real-world data, Guideline-based therapy, Antibiotic

## Abstract

**Purpose:**

Real-world data on antibiotic management of nontuberculous mycobacterial lung disease (NTM-LD) is limited for many countries. This study aimed to evaluate real-world treatment practices of NTM-LD in the Netherlands using medication dispensing data.

**Methods:**

A retrospective longitudinal real-world study was conducted using IQVIA’s Dutch pharmaceutical dispensing database. The data are collected monthly and include approximately 70% of all outpatient prescriptions in the Netherlands. Patients initiated on specific NTM-LD treatment regimens between October 2015 and September 2020 were included. The main areas of investigation were initial treatment regimens, persistence on treatment, treatment switching, treatment compliance in terms of medication possession rate (MPR) and restarts of treatment.

**Results:**

The database included 465 unique patients initiated on triple- or dual-drug regimens for the treatment of NTM-LD. Treatment switches were common and occurred approximately 1.6 per quarter throughout the treatment period. The average MPR of patients initiated on triple-drug therapy was 90%. The median time on therapy for these patients was 119 days; after six months and one year, 47% and 20% of the patients, respectively, were still on antibiotic therapy. Of 187 patients initiated on triple-drug therapy, 33 (18%) patients restarted antibiotic therapy after the initial treatment had been stopped.

**Conclusion:**

When on therapy, patients were compliant with the NTM-LD treatment; however, many patients stopped their therapy prematurely, treatment switches often occurred, and part of patients had to restart their therapy after a longer treatment gap. NTM-LD management should be improved through greater guideline adherence and appropriate involvement of expert centers.

## Introduction

Nontuberculous mycobacteria (NTM) are important causative agents of opportunistic infections [1]. In susceptible individuals, NTM can cause infections in the lungs (NTM-LD) (which is the most common presentation of NTM disease), lymph nodes, skin, and/or soft tissues or multiple organ systems (disseminated disease) [2]. NTM-LD affects susceptible patients with underlying lung conditions and/or immunological defects including disease and medical interventions [3]. NTM-LD may impact a patient’s life substantially and is associated with impaired quality of life, lung function decline, radiological deterioration and increased risk of mortality [4].

Treatment of NTM-LD is challenging and encompasses administration of multidrug regimens for a long duration of time [5]. The guidelines on the practical management of NTM-LD have been recently updated and focus on the most common NTM species causing NTM-LD [5]. Despite this guideline, its previous version and other guidelines and consensus documents in this setting, treatment outcomes are unsatisfactory [3, 6, 7].

Moreover, several studies have reported low adherence to guidelines by healthcare professionals managing NTM-LD patients in the US, Japan and European countries [8, 9]. The results of these survey-based studies indicate suboptimal treatment of NTM-LD, including inappropriate drug regimens or drug combinations or therapy duration with a potential risk of acquired antibiotic resistance. This suboptimal treatment likely contributes to unsatisfactory treatment success rates and, subsequently, poor patient outcomes [10–12]. Furthermore, treatment pattern studies using health care claims data in Germany and Japan have shown even lower rates of appropriate multidrug regimen use for the NTM disease management [13–15]. In Germany, less than 50% of patients received guideline based therapy [16]. In Japan, 55% of the treated patients received non-standard NTM-LD treatment [14].

Currently, specific information about NTM disease management in daily practice in the Netherlands is lacking. Therefore, this study aims to evaluate recent treatment practices by utilizing a Dutch pharmacy dispensing database, focusing primarily on treatment regimens used for NTM-LD.

## Materials and methods

To meet the study objective, a retrospective longitudinal real-world study was conducted on patients receiving treatments for NTM-LD in the Netherlands.

Five main topics were investigated:

1. Treatment initiation: number of patients initiated on different drug combinations for NTM-LD.
2. Treatment switches: number of treatment switches following the initial drug regimen.
3. Treatment compliance in terms of medication possession rate (MPR) defined as the days’ supply of medications in a specific period (here defined by the date of first prescription and end of the last prescription) divided by the number of days in the period. MPR represents the degree to which a patient conforms to a prescribed course of medication (e.g. MPR of 90% means that a patient has conformed to the prescribed therapy 90% of the time). MPR considers a given time period and, based on the dispensed quantity of medication, evaluates whether the patient has in his/her possession sufficient medication to optimally adhere to the expected treatment regimen.
4. Treatment persistence: continuation of treatment over time and mean therapy duration.
5. Treatment restart rate: defined as the event of having stopped NTM-LD treatment for more than 2 months and subsequently restarted therapy.

### Data

Data analysis was conducted on the data collected in IQVIA’s Dutch pharmaceutical dispensing database. The data are collected from retail pharmacies and hospitals’ outpatient pharmacies in the Netherlands. The data are collected monthly and include approximately 70% of all outpatient prescriptions in the Netherlands. Data were included from October 2014 to September 2020, with the first 12 months of data used as a look-back period to define treatment-naïve patients. Patients were followed over time and across pharmacies through a unique patient ID. The following data fields were included in this study: year of birth, sex, prescription date, prescriber specialty, dispensed medication, medication dosage and strength, and duration of the prescription (e.g., 28 days).

### Inclusion and exclusion criteria

The following inclusion criteria were applied:

- Patients of 18 years and older initiated on NTM-LD treatment (as defined in this study as the triple- and dual-drug treatment combinations presented in Table 1) between October 2015 and September 2020.

The following exclusion criteria were applied:

- Patients whose NTM-associated medication was prescribed by a dermatologist. It was assumed that these patients suffered from NTM skin infection, rather than NTM-LD.
- Patients with a total NTM-LD therapy duration of fewer than 14 days.
- Patients who were initiated on a regimen of a macrolide monotherapy for at least 1 month, where the following combinations were added: rifampicin (with or without isoniazid) for a duration of 3–4 months; isoniazid for 6–9 months. These regimens are associated with treatment for latent tuberculosis infection. Here, the latter was presumed to have developed in addition to an existing and separate health condition that had already been treated with azithromycin monotherapy.
- Patients who were initiated on a regimen of a macrolide monotherapy for at least 1 month, where the following drug combinations are added: rifampicin, pyrazinamide and ethambutol; isoniazid, rifampicin, and ethambutol or isoniazid, rifampicin, pyrazinamide and ethambutol, and where all combinations were given for 6–9 months. These patients are presumed to much more likely have a specific indication requiring macrolide monotherapy, and a new, separate diagnosis of active tuberculosis infection.

**Table 1:** Treatment combinations considered to be NTM-LD treatments

Triple-drug combinations for treatment of NTM-LD (primary analysis)	Dual-drug combinations possibly prescribed for the treatment of NTM-LD (exploratory analysis)
▪ Azithromycin + ethambutol + rifampicin ▪ Azithromycin + ethambutol + rifabutin ▪ Azithromycin + ethambutol + clofazimine ▪ Clarithromycin + ethambutol + rifampicin ▪ Clarithromycin + ethambutol + rifabutin ▪ Clarithromycin + ethambutol + clofazimine	▪ Azithromycin + rifampicin ▪ Azithromycin + rifabutin ▪ Clarithromycin + rifampicin ▪ Clarithromycin + rifabutin ▪ Azithromycin + ethambutol ▪ Clarithromycin + ethambutol ▪ Azithromycin + clofazimine ▪ Clarithromycin + clofazimine

### Assumptions

Given that IQVIA’s database on which the analyses are conducted consists of real-world data not specifically collected for this study, several assumptions were made (Table 2).

**Table 2:** Assumptions made for the analyses

Treatment initiation
▪ Patients meeting the inclusion criteria of using any of the triple-drug combinations listed in Table 1 were assumed to have received this combination to treat NTM infection, except in the cases described in the exclusion criteria. ▪ Patients meeting the inclusion criteria of using any of the dual-drug combinations listed in Table 1 were assumed to potentially have received this combination to treat NTM infection, except in the cases described in the exclusion criteria. ▪ Patients not using any of the treatment combinations of Table 1 within one year prior to the initiation were considered to be newly diagnosed with NTM and newly initiated on NTM treatment.
**Treatment switches**
▪ If patients picked up a different drug combination from the list in Table 1 than the initial therapy, they were considered to have switched therapy.
**Treatment persistence and restart of therapy**
▪ Patients with a treatment gap exceeding 1 month were considered to have stopped treatment ▪ A restart of treatment was reported based on two intervals: restart within 12 months after a treatment gap of > 2-month period, and restart with a treatment gap exceeding 12 months. In the latter case, it was assumed that a new NTM infection has occurred and patients were counted as newly initiated patients.

Data analyses were conducted in SAS (version 9.3) and Microsoft Excel.

Although international guidelines for the management of NTM-LD recommend using triple-drug combinations [5], dual-drug combinations have been used in clinical practice to treat NTM-LD [14]. Considering that dual-drug combinations could also be used for extrapulmonary NTM disease (e.g., skin infections with NTM) or that individual drugs considered here in dual-drug regimens are prescribed for non-NTM conditions (e.g., latent TB), results referring to dual-drug regimens were included as exploratory analyses, with primary analysis on triple-drug regimens that are NTM-LD-specific.

### Ethical considerations

All the research activities were performed in accordance with the declaration of Helsinki.

The study did not include medical records or human tissue; data involved was extracted from a database fully anonymized. Therefore, this study does not require ethical approval and was therefore waived by the national regulations – Central Committee on Research involving human Subjects (CCMO).

## Results

### Treatment initiation

**Fig. 1:**
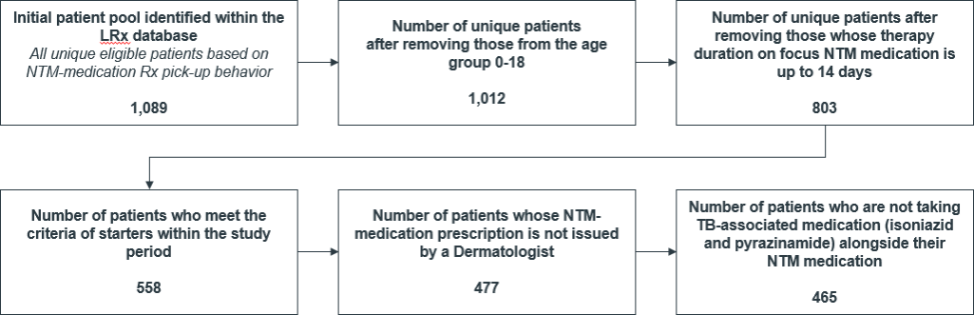
Identification of study patient population (flow diagram)

As shown in Fig. 1, between October 2015 and September 2020, the database included 465 unique patients initiated on triple- or dual-drug regimens. These patients were newly initiated on these therapy combinations 482 times (this means that some patients restarted an NTM-LD therapy after a treatment gap of 12 months or more, which was considered as a newly initiated treatment). In 187 cases the treatment initiation involved a triple-drug regimen, and 295 cases involved initiation on a dual-drug regimen. Patient characteristics and distribution across initial therapy regimens are presented in Tables 3 and 4, respectively.

The most prescribed triple-drug therapy was azithromycin + ethambutol + rifampicin and the most prescribed dual-drug therapy was azithromycin + rifampicin. Triple-drug therapy was more often prescribed in patients > 60 years old, and dual-drug therapy appeared more often in patients of the age ≤ 60 years (Table 3). For most of the patients, treatment was initiated by the pulmonologist (36.1%), the GP (18.5%) or the internal/infectious disease specialist (13.3%) (Table 5).

**Table 3:** Patient characteristics

	Patients initiated on triple-drug therapy (assumed for NTM lung infections)	Patients initiated on dual-drug therapy (potentially for NTM lung infections)	Total
N	187	295	482
Age at initiation of therapy			
Mean (SD)	61.56 (13.93)	50.23 (18.31)	54.63 (17.62)
18–29	5 (3%)	56 (19%)	61 (13%)
30–39	11 (6%)	36 (12%)	47 (10%)
40–49	15 (8%)	43 (15%)	58 (12%)
50–59	39 (21%)	53 (18%)	92 (19%)
60–69	62 (33%)	60 (20%)	122 (25%)
70–79	43 (23%)	31 (11%)	74 (15%)
≥ 80	12 (6%)	16 (5%)	28 (6%)
Female	87 (47%)	155 (53%)	242 (50%)
Male	100 (53%)	140 (47%)	240 (50%)

**Table 4:** Number of patients initiated on triple- and dual-drug therapy between October 2015 and September 2020

	18–40 years	41–60 years	> 60 years	Total
**Triple-drug therapy**	**17**	**59**	**111**	**187**
Azithromycin + Rifabutin + Ethambutol	2	4	2	8
Azithromycin + Rifabutin / Rifampicin + Ethambutol^a^			1	1
Azithromycin + Ethambutol + Rifampicin	9	42	75	126
Clarithromycin + Rifabutin + Ethambutol	4	3	7	14
Clarithromycin + Ethambutol + Rifampicin	2	10	26	38
**Dual-drug Therapy**	**98**	**100**	**97**	**295**
Azithromycin / Clarithromycin + Rifampicin		1	1	2
Azithromycin + Rifabutin	1	1	2	4
Azithromycin + Ethambutol	2	10	28	40
Azithromycin + Rifampicin	72	51	33	156
Azithromycin + Clofazimine	1			1
Clarithromycin + Rifabutin	3	1	1	5
Clarithromycin + Ethambutol	4	20	20	44
Clarithromycin + Rifampicin	14	16	12	42
Clarithromycin + Clofazimine	1			1
**Total**	**115**	**159**	**208**	**482**

^**a**^*Note*: *In the context of this research, rifabutin and rifampicin, and azithromycin and clarithromycin, are considered interchangeable medications, even if there is some overlap observed in the database, as they are not expected to be administered together for the treatment of NTM-LD.*

**Table 5:** Initiating specialty of triple- and dual-drug therapy between October 2015 and September 2020

Dual- and triple-drug therapy		Triple-drug therapy		Dual-drug therapy	
Initiating specialty	Percentage of total	Initiating specialty	Percentage of total	Initiating specialty	Percentage of total
Pneumologist	174 (36.1%)	Pneumologist	125 (66.8%)	General practitioner	77 (26.1%)
General practitioner	89 (18.5%)	Internist	28 (15.0%)	Pulmonologist	49 (16.2%)
Internist	64 (13.3%)	General practitioner	12 (6.4%)	Internist	36 (12.2%)
Other/unknown	155 (32.2%)	Other/unknown	22 (11.8%)	Other/unknown	133 (45.1%)

### Treatment switches

**Fig. 2:**
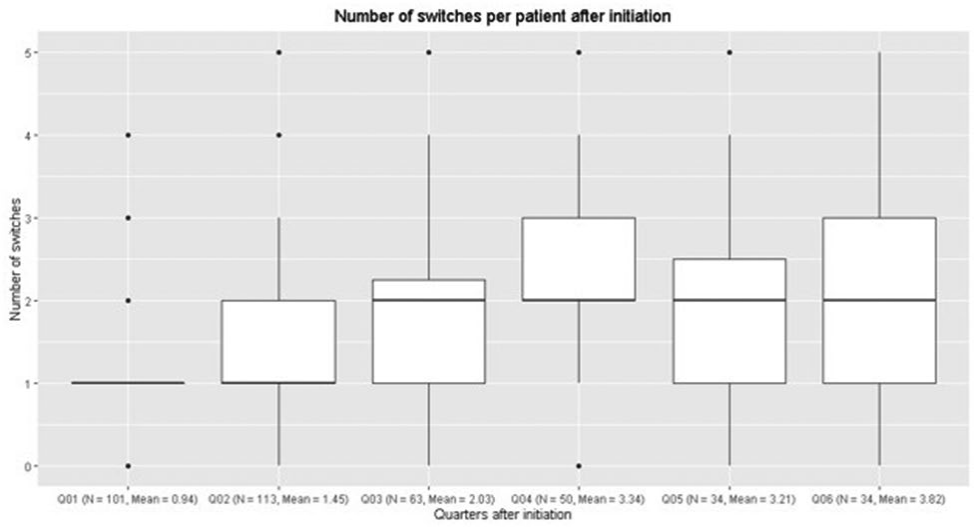
Treatment switches per three months for patients initiated on triple-drug regimen

**Fig. 3:**
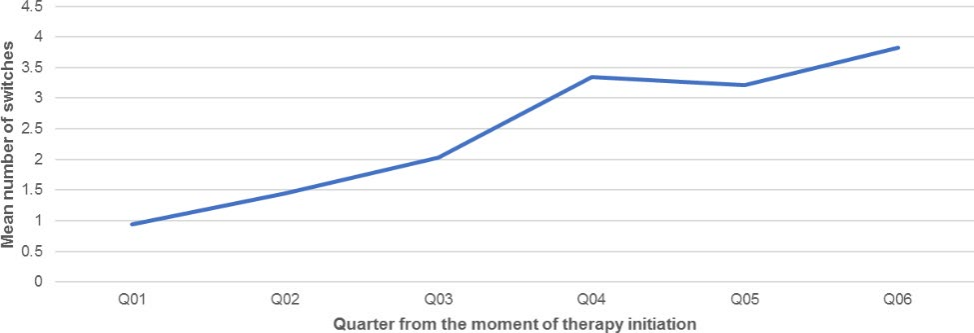
Mean cumulative number of switches after therapy initiation

Figures 2 and 3 show the number of switches per patient per consecutive quarter and the mean cumulative number of switches from the moment they were initiated on triple-drug NTM therapy, respectively. As can be seen, the number of patients declines over the quarters, but the number of mean switches remains relatively stable at approximately 1.6 switches per quarter. Some therapy switches were temporary. In such a case, one or more drugs were temporarily stopped and added again later. The most commonly observed switches amongst triple-therapy starters were from “Azithromycin + Ethambutol + Rifampicin” to “Azithromycin + Rifampicin” (47 unique switches within the study period); from “Azithromycin + Ethambutol + Rifampicin” to “Ethambutol + Rifampicin” (39 unique switches); from “Azithromycin + Ethambutol + Rifampicin” to “Ethambutol” and “Azithromycin + Ethambutol” (38 unique switches each).

The unique instances of patients switching from a regimen including Azithromycin to a regimen including Clarithromycin, and vice versa, are 25 in total.

The number of switches was comparable between patients up to 60 and above 60 years of age. Patients starting on dual-drug therapy showed similar therapy switch behavior.

### Treatment compliance

The average MPR of patients initiated on triple-drug therapy was 90%. Compliance did not differ much between different triple-drug combinations (Table 6). The MPR of patients up to the age of 60 was similar to that of patients above 60 years of age.

**Table 6:** Medication possession ratio while on triple-drug therapy combinations

Triple-drug therapy	N	Mean (SD)
Azithromycin + Rifabutin + Ethambutol	8	90% (19)
Azithromycin + Rifabutin / Rifampicin + Ethambutol	1	100%
Azithromycin + Ethambutol + Rifampicin	126	91% (17)
Clarithromycin + Rifabutin + Ethambutol	14	92% (17)
Clarithromycin + Ethambutol + Rifampicin	38	93% (15)
Total	187	91% (17)

### Treatment persistence for patients on triple-drug therapy

**Fig. 4:**
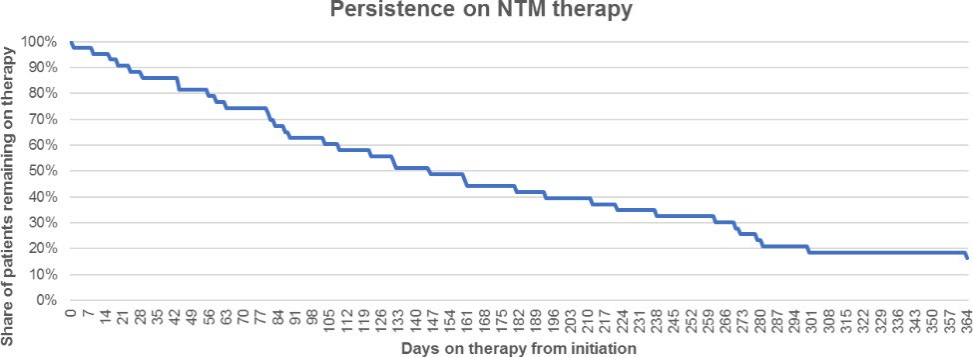
Treatment persistence of patients starting on NTM triple-drug therapy

Treatment persistence was analysed for patients initiated on triple-drug therapy between October 2015 and September 2016 (N = 43). The median time on therapy for these patients was 119 days. The median therapy duration of the patients who completely stopped was 105 days, while that of patients who discontinued one or two antimycobacterial drugs was 153 days. After six months, 47% of the patients were still on therapy, and after one year – approximately 20% (Fig. 4). Moreover, patients remaining on therapy often discontinued one or two drugs of their initial regimen (Table 7).

**Table 7:** Treatment discontinuation for patients initiated on triple-drug therapy

Quarterly periods	Total number of patients still on therapy	From those, patients still on triple-drug therapy	From those, patients discontinuing one or two drugs of the triple-drug therapy regimen but remaining on at least one of the drugs from the triple-drug combination
0 – patient pool upon initiation	43	15	28
3 months	27	8	19
6 months	19	7	12
9 months	13	6	7
12 months	8	3	5

### Restart of therapy

Of all patients initiated on triple-drug therapy (N = 187), 27 (14%) patients had a restart of therapy within 2–12 months after the treatment had been stopped and 5 patients (3%) had a restart after more than 12 months following initial treatment termination. Most of the patients who restarted, restarted with a triple-drug regimen (27), while the remainder (5) restarted with a dual-drug combination. The average time between stopping and restarting for patients who restarted after > 12 months was 952 days, while the average gap for patients who restarted within 2–12 months was 118 days.

A similar proportion of patients that were initiated on dual-drug therapy underwent a restart of NTM therapy after at least 2 months of a treatment gap (N = 44/295; 14.9%). Only 8 of those patients restarted with a triple-drug regimen, while the remainder restarted with a dual-drug combination.

## Discussion

In this study, real-world treatment patterns in patients with NTM-LD in the Netherlands were investigated using medication dispensing data, specifically looking at a selection of initial treatment regimens, persistence on treatment, treatment switching, compliance with treatment, and finally restart of treatment.

The most striking observation in this study is the very low total therapy duration. Total therapy duration for NTM-LD is determined depending on sputum culture results. It is pivotal and recommended by international guidelines that treatment continues 12 months after sputum culture conversion. Given the fact that most patients show conversion within 6 months, therapy duration for NTM-LD, in general, should last up to 18 months [16]. In our study, the median treatment duration was approximately 4 months. After half a year, only 47% of the patients were still on triple-drug therapy and after one year this dropped to 19%. This is comparable to the finding from a study performed in Germany by Diel et al. [15] where it was reported that the antibiotic therapy lasted for at least 6 months in 42%, for at least 12 months in 24% of patients. This observation is dramatic since most patients are faced with serious morbidity and mortality especially for certain NTM-LD phenotypes (mainly fibrocavitary disease). The therapy duration observed in this study may reflect uncertainty about the necessity of the use of antibiotics in patients with the nodular-bronchiectatic NTM-LD phenotype that have a much more indolent course. Particularly these patients may potentially struggle with toxicity and tolerance issues dominating over (mild) symptoms related to the NTM infection. This low treatment persistence can sometimes have a clinical reason, such as poor tolerability, or drug interaction due to polypharmacy. However, in many cases, it could also be the cause of poor patient compliance to long-term therapy or the lack of experience by physicians with regards to managing these patients appropriately. Therefore, it is crucial that much more attention is being paid to educating and engaging both patients and physicians.

In addition to the previous concerns, approximately 1 in 6 patients restarted therapy after a treatment stop of at least 2 months, which can indicate disease recurrence due to a relapse or a new NTM infection. Reoccurrence could be likely as a result of the short treatment duration observed in this study [6, 17]. Disease recurrence is common in these patients – previous studies reported that 10% to up to approx. 50% of patients with NTM-LD who are initially treated successfully have a disease recurrence [10, 17–20]. These rates of recurrence vary considerably as they depend on characteristics of the patient cohort studied, the environmental exposure and possible difference in virulence of the NTM species involved. Our study analysed treatment re-initiation and not a microbiological outcome, thus direct comparison to other studies is somewhat hindered. And still, drug intolerances can also be an important explanation of an observed stop and restart case. Better adherence to treatment guidelines (especially regarding treatment duration) may decrease disease recurrence. A recent meta-analysis showed that treatment success is indeed better when all the American Thoracic Society criteria are in place [10].

It was observed that initial dual-drug treatment regimens were more commonly prescribed than guidelines-based triple-drug regimens. Note that it is possible that not all instances of dual-drug therapy found in this study are NTM-LD prescriptions. This may be the case, especially, where dual-drug therapy was prescribed by internal medicine specialists and GPs. Importantly, patients treated by Dermatologists with dual- or triple-drug regimens were excluded from the study to screen out possible cases of NTM skin and soft tissue infections. These results (and the results regarding the length of treatment) indicate adherence to the guidelines may not be optimal, a finding that was also observed in previously published studies from the US, Europe and Japan [8, 9, 13, 14, 21–24]. Another possible explanation is that physicians decide about the regimen depending on the results of incorrectly performed antimicrobial susceptibility testing, although it should be noted that the practice of incorrect testing is disappearing in the Netherlands. Rifampicin and ethambutol show very poor in vitro activity against most *Mycobacterium avium* complex (MAC) species, the most prevalent NTM species causing NTM-LD; if breakpoints for resistance of *Mycobacterium tuberculosis* are applied, this could be interpreted and reported as resistance [25]. Therefore, in certain cases, the physician may decide to omit one of these drugs and initiate dual-drug therapy. However, these drugs do not have a role in mycobacterial killing but are crucial in preventing the occurrence of macrolide resistance. Susceptibility testing and reporting for these drugs are not indicated and current guidelines state it should not be done [26, 27]. The troublesome finding is that the most prevalent dual-drug therapy observed in our study (rifampicin plus macrolide) may increase the risk of developing macrolide resistance in the case of NTM-LD caused by MAC [8].

This is the first study to report on patient compliance with treatments for NTM-LD through prescription data in the Netherlands. We found high rates of medication possession ratio (> 90%) for patients on various treatment regimens. The approach used was based on prescription refills over certain time-interval which is one of the common methods to measure treatment compliance [28]. This allows assessing (non)compliance in a large population over an extensive period. Note that the medication possession rate shows whether patients pick up their medication and their prescription refills, but it does not show whether the medication was taken. Consequently, compliance rates may be overestimated [28]. Considering that we observed on the one hand high drug compliance rates, meaning that patients were likely to have been taking their medication regularly, and on the other hand early treatment discontinuation, it would be of interest to further explore the reasons why therapy is being prematurely terminated.

Another notable fact is that treatment switches frequently occur throughout the treatment period. In each consecutive quarter, one-third to half of the patients who were still on therapy switched treatment (e.g., from dual- to triple-drug therapy, the other way around, or switched to another triple- or dual-drug therapy), with approximately one treatment switch per half-year. Note that some therapy switches were temporary. In such a case, patients stopped one or more drugs of the drug combination temporary and started again later. Frequent therapy switches observed are not surprising, considering that chances of achieving treatment success are suboptimal [10, 11] and high rates of drug toxicity are observed in clinical practice [29]. Therefore, for patients escalating from dual- to triple-drug therapy, the switch may have been done due to their clinical deterioration or following a second opinion. Replacing specific medication or switching from triple- to dual-drug regimen is however likely to be because of drug toxicity issues; in this complicated setting, little guidance is available but switching drug classes is generally preferred over stopping one drug of the triple-drug regimen [26]. Results of antimicrobial susceptibility testing may also impact regimen composition, despite only being warranted for macrolide resistance. Moreover, switching from triple-drug therapy to ethambutol monotherapy for a while before resuming with triple-drug or another regimen may be a strategy to assess the cause of drug-related adverse events by the treating physician. However, this hypothesis cannot be confirmed with the current data.

An asset of this study is that the percentage of national coverage of the pharmacy dispensing database is high (approx. 70%). The generalizability of the outcomes of this study for the Dutch setting is therefore relatively good. The most important limitation of this study is that the database does not include clinical diagnoses and treatment indication was not available. Consequently, the study assumed that specific drug prescriptions in the database were prescribed for NTM-LD. Although the triple-drug combination on which our primary analyses were based is very likely to be specific for NTM-LD, the dual-drug regimens were, for this lack of disease codes, used for exploratory analyses only. Regarding the MPR, the data in this study does not provide information about patients who were prescribed medication but did not pick it up. In this study, such instances are categorized as a therapy gap or treatment stop instead of a possible compliance problem of the patient.

## Conclusion

This is the first study to provide insights into the real-world treatment of NTM-LD in the Netherlands using medication dispensing data. The study found that when on therapy, patients were compliant with the treatment, however, many patients stopped their therapy prematurely, treatment switches often occurred, and part of patients had to restart their therapy after a longer treatment gap. A restart of treatment was frequently observed and was likely associated with the short therapy duration that was also found in this study. NTM-LD treatment and consequently treatment outcomes could thus be improved through improving guideline adherence and appropriate involvement of expert centers.

## Data Availability

The datasets generated and/or analysed during the current study are not publicly available. The data are available upon request from the corresponding author upon reasonable request.
